# The Importance of Consumer Authorities for the Production and Maintenance of Trust and Social Capital in Consumer Markets

**DOI:** 10.1007/s10603-022-09523-6

**Published:** 2022-07-04

**Authors:** L. Berg

**Affiliations:** SIFO – Consumption Research Norway, Oslo Metropolitan University, Oslo, Norway

**Keywords:** Consumer trust, Generalized trust, Social capital, Institutionalized distrust, Consumer conditions, Consumer markets

## Abstract

Trust is a valuable resource that varies between countries. This paper suggests that consumers’ trust in retailers and service providers, facilitating interactions and transactions between sellers and buyers in impersonal markets, is best understood as *generalized trust*. The paper is based on 28 037 respondents’ evaluations of consumer conditions in 30 European countries. The material reveals large country-to-country variations in the percentages of residents who trust public authorities to protect their consumer rights. Moreover, there are large differences in the percentages who trust retailers and service providers to respect their rights as consumers. A multilevel path analysis supports the paper’s main hypothesis that *fair and effective consumer authorities enhance generalized trust in the markets.* The analyses also demonstrate that fair and effective consumer institutions contribute to more equality in the markets. It is argued that consumer markets are important arenas for the maintenance and production of trust and social capital. And that generalized trust produced in markets will probably extend to, and be valuable for, the wider society.

This paper focuses on consumer markets and suggests that consumers’ trust in retailers and service providers should be considered as one specific kind of *generalized trust* appearing in a specific setting, namely in interactions and transactions between buyers and sellers in impersonal consumer markets.

It is widely acknowledged that generalized trust—the belief that people in general can be trusted—is beneficial for citizens’ wellbeing, social integration, economic growth, and democracy (Alecu, 2020; Algan & Cahuc, 2013; Bjørnskov, 2012; Granovetter, 1985; Hooghe & Stolle, 2003; Putnam, 1993, 2000, 2001; Rothstein & Stolle, 2008). Trust facilitates cooperation and reduces transaction costs (Fukuyama, 1995) and enables relations between people within societies, economies, and organizations (Noteboom, 2002). Interpersonal trust and trust in government were found to correlate with COVID-19 vaccine uptake and reduced infection-fatality (The Lancet, 2022). According to Elster (1989), trust can be described as a social lubricant, without which the wheels of society will soon come to standstill. Accordingly, how generalized trust is produced and maintained deserves attention.

In the OECD countries, household expenditures on consumption typically represent about 60% of GDP (OECD, 2022). In one year, almost every European visits the food markets, as well as the clothing and shoe markets (European Commission, 2018, p. 73). The average time that Europeans between the ages of 20 and 74 spend shopping for goods (such as visiting a grocery store) and for services (such as going to a hairdresser) ranges from 17 min *per day* in Romania to 35 min in Germany (Eurostat, 2018). So, markets are certainly important arenas for citizens’ interactions and transactions with other—often unknown—people.

Every little transaction in a market, with the possibility of being satisfied or disappointed, has the potential to influence trust in that provider and/or that product (Choi & Storr, 2020). It is plausible, then, to assume that a person’s overall experiences from the markets will influence that person’s generalized trust.

Trust has been described as a complexity-reducing mechanism that makes life easier (Luhmann, 1979). Coleman (1988, 1990) considers trust and trust relations as *social capital*, a valuable resource at both the individual and the societal level. And, according to Fukuyama (1995, 2002), societies rich in social capital, understood as cultures of trust, facilitate cooperation among people. Leaning on insights from these works, safe consumer conditions can be described as a web of interpersonal trust relations establishing cultures of trust, i.e., social capital, that makes the consumer role easier to perform, hence contribute to simpler life for the individual citizen. At the societal level, consumer markets characterized by trust are advantageous in terms of more effective market transactions, which benefits the economy and the wider society (Coleman, 1988, 1990; Fukuyama, 1995, 2002; Luhmann, 1979).

According to Rothstein (1998), *political institutions* structure decision-making situations and *influence trust* by implementing norms and regulations. Referring to North (1990), Rothstein stresses that, because *we can choose how to design our political institutions*, we can to some extent decide which norms will prevail in the society we live in. Institutions can change not only what actors regard as rational actions, but also what they regard as morally correct actions. It follows that the design of consumer policy institutions—including consumer laws—will affect norm-governed behaviour in markets and thereby affect consumer conditions and trust.

By addressing the role of consumer institutions in promoting safe consumer conditions and trust, the contribution of this paper is to make more explicit the importance of *institutionalized distrust*—i.e., consumer institutions’ critical-reflective activities—for the production and maintenance of *interpersonal* *trust* in the markets. And not the least, by introducing consumer markets as vital arenas for the production and maintenance of generalized trust, the following analyses aspire to add some empirical evidence to the role institutions play in the development of generalized trust (Berggren & Jordahl, 2006; Rothstein, 1998, 2011; Rothstein & Stolle, 2008; Sztompka, 1998, 2010) and social capital (Coleman, 1988, 1990).

First, two subsidiary hypotheses are tested, one at the societal level and one at the individual level, respectively:

- *Fair and effective consumer authorities (and their executing institutions) enhance safe consumer conditions.*
- *Trust in consumer authorities affects individual generalized trust in retailers and service providers.*

Then the main hypothesis, including variables at societal and individual levels, is tested:

- *Fair and effective consumer authorities enhance generalized trust in the markets.*

These hypotheses are investigated using data collected by the European Commission for its annual Consumer Scoreboards (European Commission, 2019). Two other papers utilizing prior Consumer Conditions Scoreboard materials (Berg, 2014; Nessel, 2021) approached similar research questions concerning trust in markets, but only at aggregated country levels. This paper takes these analyses one step further, analysing the Consumer Conditions Scoreboard material at both aggregated *and* individual levels. Specifically, it looks at how 28 037 respondents living in 30 different European Countries, with differently designed consumer institutions, evaluate their consumer conditions, as well as how consumer institutions affect individual generalized trust. This paper does not intend to discuss or give an overview of the many ways that trust has been approached, understood, and conceptualized (Cook et al., 2005; de Jager, 2017; Hobbs & Goddard, 2015; Luhmann, 1979, 1988; Misztal, 1996; Nooteboom, 2002). Rather it responds to Fukuyama’s (2002) request for a more pragmatic approach to the understanding of trust and social capital, by, e.g., examining empirically the origins of social capital.

The paper is divided into four sections. The “Background” section describes the analytical model, relevant literature, and concepts. The next section presents the data and methodological approaches. The “Results” section begins by mapping the main variables. Next, it offers a regression analysis at *society level*, where the units are the countries included in the material. Then, to search for robust patterns, results from 26 multivariate regression analyses, at *individual level*, from 26 different countries, are listed and compared. Lastly, a *multilevel* path analysis is estimated. The last section of the article discusses the results and their significance.

## Background

This paper draws attention to the importance of consumer authorities and their executing institutions for well-functioning markets, seen from the consumer’s perspective. According to traditional economic theory (Smith, 1776), well-functioning markets primarily depend on fair competition among suppliers. Consumers are expected to make rational choices in the markets, assuring a healthy balance between consumer demand and seller supply. Many social scientists have criticized this theory as too simplistic, neglecting problems on the consumer side of the market forces, such as bounded rationality (Simon, 1957, 1982), the information paradigm (Stiglitz, 2002), the capability approach (Sen, 2009), the importance of norms (Elster, 1989, 1990), cultures of trust (Fukuyama, 1995), and the consumer attention-deficit problem (Berg & Gornitzka, 2012). Contrary to traditional economists, behavioural economists stress that consumers are humans, not (always) rational actors, and they point to cognitive mechanisms within individuals to explain widespread and predictable non-rational choices and behaviours (Kahneman, 2003, 2011). Thaler and Sunstein (2008) show how insights from behavioural economics can be used by governments and institutions to construct choice architectures that “nudge” people in preferred directions. Governments, consumer authorities, and policymakers worldwide are now taking advantage of insights from behavioural economics to improve consumer conditions (European Commission, 2022a; Reisch & Zhao, 2017).

Accordingly, the following study relies on the view that well-functioning markets depend on *three parties* playing their roles in satisfactory ways. On the demand side, *the consumers*’ role is to make active choices in the marketplace, voting with their wallets for the products they want to remain on store shelves and complaining when they have legitimate reasons to do so (Berg & Gornitzka, 2012; Hirschman, 1970), so that *retailers and providers*, who compete for consumers’ attention and money, will produce and bring to the markets the things that consumers need and want (Smith, 1776). In Western democratic welfare states, *public authorities*, acting within their institutions, monitor the markets, setting rules and ensuring that retailers and suppliers follow competition and consumer laws, thereby helping retailers and suppliers and consumers to play their roles in ways that benefits everyone in the society (European Commission, 2022b, c).

### Trust, Generalized Trust, and Social Capital

It is commonly agreed that *trust* is central to the well-functioning of consumer markets (Arrow, 1972; Coleman, 1988; European Commission, 2018, 2019; Granovetter, 1985; Misztal, 1996). There is little agreement, however, as to how trust should be approached, understood, and conceptualized. Nooteboom (2002) defines trust as a (reflected) rational evaluation of the evidence for trustworthiness, whereas Giddens (2003) states that (unreflected) familiarity is the keynote of trust. Luhmann (1988) includes both perspectives and distinguishes among “trust,” “confidence,” and “familiarity,” with each successive term implying decreasing acknowledged risk and reflectivity. In this paper, *generalized consumer trust* is understood as *consumers’—more or less reflected—considerations as to how reliable retailers and suppliers in general are.*

To comprehend fully the value of generalized trust, it is helpful to consider this concept in relation to *social capital*. Coleman (1988) was probably the first social scientist to use the term social capital. His broad idea was that social relationships characterized by trust and trustworthiness are *resources*—other than financial, physical, and human capital—that help people act efficiently. According to Coleman (1990), social capital differs from other forms of capital as it is simultaneously a private and public good. There is broad agreement that generalized trust and social capital are beneficial at both the individual and the societal level (Alecu, 2020; Arrow, 2000; Berggren & Jordahl, 2006; Dasgupta & Serageldin, 2000; Putnam, 1993, 2000).

Like for the concept of trust, however, there is little consensus on how generalized trust and social capital should be understood and defined (Alecu, 2020; Claridge, 2020; Nannestad, 2008). Sometimes generalized trust and social capital are used synonymously, other times employed as overlapping, or independent concepts (Alecu, 2020). In this paper, the two terms are understood as two sides of the same coin, mutually supportive in a dialectical relationship. On the one side, *generalized trust* here refers to consumers’ general trust in the suppliers with whom they may come to interact with in the markets. It facilitates future (trustful) interactions and transactions, unless bad experiences give reason for the withdrawal of trust. On the other side of the coin, *social capital* refers to a consumer’s supportive network of (trusted) family, friends, and acquaintances, including suppliers, and probably reliable institutions, that a consumer can turn to and ask for advice and support, perhaps to make better choices in a market she/he is unfamiliar with. Such networks, almost like safety nets, implicitly empower and protect individual consumers when they engage in transactions and interact in the marketplace. Overall, generalized trust and social capital together function as a resource in as much as they make the consumer role easier to perform.

Following Alecu (2020), one individual’s social capital varies according to the number of (trusted) people included in his/her network, as well as to the quality of that network determined by its members’ social and financial status, their experiences, the group’s heterogeneity, etc. More precisely, *an individual’s social capital is the set of resources—facilitating actions—someone has access to in a social network characterized by trust* (Coleman, 1988). At the collective level, a nation’s *societal capital varies according to inhabitants’ generalized trust, their well-informed participation, and not the least by the quality of its public institutions* (Marozzi, 2014). It is important to note that according to these definitions at individual and collective level, respectively, social and societal capital are interconnected by trust (Coleman, 1990).

At collective level, the World Value Survey demonstrates that there are large differences between countries in societal capital as measured by citizen’s *generalized interpersonal trust* (Nannestad, 2008). Several studies based on the World Value Survey also show that democratic countries are consistently high in measures of generalized trust, in contrast to non-democratic countries, where generalized trust is low (Sztompka, 2010; Uslaner, 2003). Many scholars regard social capital as essential to economic development and to stable liberal democracies (Fukuyama, 2002). However, in particularly one challenge, the uncertainty of the direction of causality characterizes and complicates this field of research. Are democracy and economic growth the effects or causes of generalized trust? Or is the relationship best described as virtuous and vicious circles (Nannestad, 2008)?

### Can Markets Generate Trust?

There is a common understanding that trust is the key ingredient in almost all market transactions and interactions (Arrow, 1972; Coleman, 1988; Granovetter, 1985; Misztal, 1996). There is less agreement as to whether interactions in the marketplace can generate trust in the wider society or, on the contrary, cynicism and distrust. On the one hand, some have warned that the logic of market interactions, characterized by self-interest and competitive behaviour, can infect and harm social relationships, particularly when expanding markets lead people to spend increasingly more time in the marketplace and to adapt to its logic (Etizoni, 1988; Gray, 1998; Gudeman, 2001; Weber, 1921). On the other hand, some have shed light on market-based interactions and transactions that appear to generate interpersonal trust and even friendship (Choi & Storr, 2020; Dulsrud & Grønhaug, 2007). Berggren and Jordahl (2006) argue that institutions promoting a well-functioning-free economy reinforce a climate of trust and that well-functioning markets can be expected to give rise to generalized trust.

This paper asserts that consumer markets are important arenas for the maintenance and production of generalized trust. However, distrust can also arise. Fraud, or unfair and unlawful practices in markets, can result in consumer detriments, dissatisfaction, and distrust (Berg, 2016; European Commission, 2017; OECD, 2020).

According to the literature, it seems as if markets can contribute both positively and negatively to the generation of interpersonal trust/distrust. One highly relevant question, then, is how markets should be organized to produce trust and not distrust? Obviously, unfair commercial practices, including fraud and sales of fake products, erode trust, whereas safe consumer conditions and good consumer experiences foster trust. The purpose of this paper is precisely to investigate whether, or not, consumer authorities and their institutions can contribute to improved market conditions as measured by safe consumer conditions, followed by higher levels of generalized trust in markets and societies.

### Consumers’ Contribution to Well-Functioning Markets

Consumers play an important role in the market game. Active and reflective consumers who give “voice” and complain, instead of offering “silent loyalty,” provide important information and correctives to the supply side (Hirschman, 1970), while unreflective consumer choices, and too many consumers failing to complain when they have reasons to do so, may stimulate undesirable market practices among suppliers (Berg & Gornitzka, 2012).

In busy people’s everyday lives, shopping habits are often quite routinized (Warde, 2015). Many other aspects of life than the consumer role fight for peoples’ attention (Berg & Gornitzka, 2012). Given these circumstances, reliable suppliers respecting consumers’ rights are gift packages facilitating the daily lives for consumers in general and in particular for the many consumers living their lives in constant time squeeze. Again, trust makes life easier (Luhmann, 1979).

Unfortunately, trust also facilitates unreflective behaviour. Trust is good—but too much trust is not constructive. Too much unreflective trust (naivety) can lead to bad consumer choices and consumer detriment (Berg, 2016). In malfunctioning markets characterized by untrustworthy actors, distrust and scepticism are safer responses than (too much) trust when consumers approach the marketplace (de Jager, 2017). The point is that, whereas *distrust stimulates to reflectivity* and activity, *trust can be passivating*, resulting in indifferent and inattentive consumer practices (Berg, 2004, 2008, 2016).

Berg and Gornitzka (2012) argue that—in the market game—consumers are often not sufficiently powerful counterparts to the suppliers. Consumers, visiting many different markets, do not possess sufficient attention capacity to keep updated on every market they visit and to always make active, informed choices and complain when there are good reasons for complaints. There is no crisis at market level if busy and trusting consumers make some unreflective and unfortunate choices. The problem arises if too many consumers make uninformed or accidental choices in the same market. If so, the supply side will not receive the feedback needed to improve or at least maintain the quality of its products. This situation can result in low-performing markets that deliver unsatisfying products (Berg & Gornitzka, 2012). And eventually, as this paper argues, it can lead to a reduction in generalized consumer trust.

### Consumer Institutions’ Contribution to Well-Functioning Markets

Such unfortunate market situations—arising when consumers are not sufficiently powerful counterparts to the suppliers—can be mitigated by effective consumer institutions performing *institutionalized distrust*, thus supporting the consumer side in the market game. According to Rothstein (2000), institutions of law and order have one important task: to detect and punish people who break contracts, steal, or do other non-cooperative things. Applied to consumer markets, the lack of reflective consumer complaints can be compensated for by institutionalized distrust performed by fair and effective consumer institutions. In a way, consumer institutions can be considered highly critical-reflective and active professional consumers.

Braithwaite (1998) outlines how institutionalizing distrust makes it easier to trust others interpersonally. Institutionalized distrust operated by accountable institutions is paving the ground for trust at interpersonal level (Braithwaite, 1998). Also, to protect democracies, Sztompka (2010) prescribes institutionalized distrust: *[D]emocracy needs both the culture of trust and the culture of distrust – but at different levels of social life. The culture of trust at the level of civil society, and the culture of distrust embedded in the institutions of government. These cultures do not contradict each other, but are mutually supportive, bound in a dialectical relationship* (Sztompka, 2010, p. 290).

Leaning on Braithwaite (1998) and Sztompka (2010), there are reasons to assume that fair and effective consumer institutions, performing institutionalized distrust, will enhance the culture of trust between buyers and sellers in consumer markets. There are also reasons to believe, that in modern complex societies, with many busy citizens visiting the markets with their minds elsewhere (Berg & Gornitzka, 2012), well-functioning markets depend more and more on institutionalized distrust performed by fair and effective consumer institutions.

In the markets, institutionalized distrust should ensure that retailers and manufacturers receive adequate corrective feedback so that they bring to the markets products that are safe to use and foods that are safe to eat. By targeting and punishing unlawful activities in the markets, while supporting serious and trustworthy suppliers, institutionalized distrust encourages well-functioning markets and safe consumer conditions, thereby supporting the consumer role and promoting consumer trust.

### Consumer Policy Across Europe

At the European level, work on consumer policy and rules of competition is driven by the European Commission (2022b, c). To stimulate the economy during the pandemic, the Commission has aimed to boost trust among consumers through measures that promote a greener, more digital, and fairer single market (*New Deal for Consumers 2020–2025*). The Commission’s emphasis on trust, as measured by both the Consumer Conditions Index and the Markets Performance Index (European Commission, 2018, 2019), indicates that consumer policymakers consider trust important for well-functioning markets.

European Union activities and legislation are strong drivers of improving consumer conditions at national levels (Nessel, 2019). The enforcement of consumer rights, however, is primarily left to national authorities, with support from consumer organizations (Austgulen, 2020). At the country level, there are two main organizational consumer policy models: state-funded institutions and volunteer organizations financed by membership fees and other sources. The BEUC, an umbrella organization of 44 independent consumer organizations from 32 countries, plays an important role in the development of consumer policy in Europe (BEUC, 2020).

The European Commission’s New Consumer Agenda, launched in 2020, stresses the growing importance of international cooperation and harmonization to ensure effective enforcement of consumer rights across Europe (European Commission, 2020). Prior to this initiative, the European Commission had already issued many consumer policy directives defining minimum consumer protection requirements for Europe (Nessel, 2019). However, diverging preferences in consumer policy across EU Member States, probably the result of different market histories, create challenges to harmonizing consumer legislation and to reach agreements on uniform measures to improve consumer conditions across Europe. Member States differ in the numbers of people and amounts of public resources they devote to consumer protection. This creates problems, such as countries with greater resources and higher levels of consumer protection expressing resistance to lowering their standards to achieve uniformity across Member States (Austgulen, 2020).

There are few comparable data on national investments in consumer institutions. Still, data collected by the European Commission’s Consumer Policy Network demonstrates that national public funding of consumer institutions differs considerably across Europe. The Nordic countries have more state-funded institutions than do other European countries (European Commission, 2015, p. 22).

Social scientists have used different, often overlapping, classifications of consumer policy regimes (Austgulen, 2020; Nessel, 2019; Repo & Timonen, 2017). Based on data collected through an open public consultation by the European Commission in 2016, supplemented by key stakeholder interviews in 2018, Austgulen (2020) looked for patterns in the interests and preferences of countries with respect to EU consumer policy. Based on prior research and classifications, she distinguished six different regimes. She concluded that each country’s position and interests with respect to consumer policy are complex but that the disparities between the six distinguished regimes are greater than are the differences within each. Drawing on Austgulen’s paper, it is possible to derive a rough understanding of differences among EU countries’ consumer protection levels. The Nordic countries (Denmark, Finland, Iceland, Norway, and Sweden), followed by the Franco-Roman countries (Netherlands, Belgium, Luxembourg, France) and the German countries (Austria, Germany), have invested more in consumer policy protection and empowerment than have Southern European (Italy, Spain, Portugal, Greece) and Eastern European countries (Bulgaria, Croatia, Czech Republic, Estonia, Hungary, Latvia, Lithuania, Poland, Romania, and Slovak Republic, Slovenia). The Anglo countries (UK, Ireland, Malta, and Cyprus) differ among themselves in regard to consumer policies. These findings support the view that consumer authorities’ capability to enforce consumers rights varies considerably across Europe.

### Analytical Model

The main purpose of the analyses that follow is to investigate how one specific kind of generalized trust*—individual generalized trust in retailers and providers* in impersonal markets—is affected by the efficiency and performance of *consumer authorities.* Are fair and effective consumer authorities, practising institutionalized distrust with the purpose to discipline the supply side, protect consumers, and reinforce consumer rights, the drivers of (1) better consumer conditions at the societal level and (2) generalized trust at the individual level? First, the dependent variable—*citizens’ perception of consumer conditions*—is considered at the national level. Then, the dependent variable—*generalized trust in retailers and providers—*is considered at the individual level. Eventually, variables at societal level and individual level are combined in a multilevel path analysis.

The consumer role is certainly affected by a person’s financial situation. For example, a country representative Norwegian survey revealed that people with limited finances (and thus limited purchasing capability) are much more likely than others to report consumer detriment measured by financial losses, fraud, and injuries from products bought on the markets, probably because safer quality products were not affordable to them (Berg, 2016). According to Sen (2009), the more capabilities—including access to and the ability to manage money—the greater are one’s opportunities, choices, and advantages. Thus, people rich in financial capabilities might have more reasons to trust, and more likely to perceive the consumer conditions in their country to be good, than might fellow citizens with limited financial capabilities. That being possible, the likelihood must be considered that a nation’s level of generalized trust is foremost determined by the financial welfare of its people, and not just by the quality of its institutions. Therefore, *citizens’ financial welfare and capability* is considered an important independent variable and controlled for at society level in the following analyses.

In addition to people’s *financial situation*, their *age*, *gender*, and *educational level* are expected to influence their roles as consumers. Older people tend to have more available time and market experiences and seem to be less vulnerable as consumers than are their younger counterparts (Berg, 2015). Male and female consumers tend to visit and master different markets with different characteristics and challenges (Berg & Teigen, 2009). Education is related to class, and self-reported class affiliation seems to affect consumer practices, preferences, and market experiences (Berg, 2019). Thus, *individual financial situation*, *age, gender,* and *education* are variables that may influence *individual generalized trust* through demographic groups’ different practices and experiences. Accordingly, these variables are controlled for at individual level in the following analyses.

**Fig. 1:**
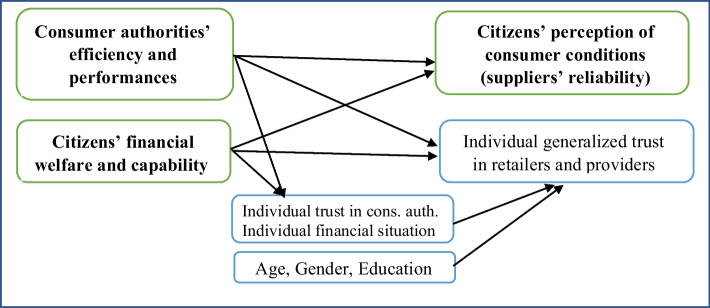
Variables at societal level (green boxes) and individual level (blue boxes) to be included in the following analyses

Figure 1 illustrates the main steps in the analyses that follow. First, with country as the unit, the effects of the two independent variables at the societal level on *citizens’ perception of consumer conditions* are estimated (boxes outlined in green, bold letters). Second, within each country, the same variables are investigated at the individual level, controlled for the group variables *age*, *gender*, and *education* (boxes outlined in blue). Eventually, all the variables affecting *individual generalized trust* are combined in a multilevel path analysis, primarily to illustrate and estimate how *consumer authorities’ efficiency and performances* affect consumers’ *individual generalized trust*.

## Methods

### Data Material

This paper builds on data collected for the European Commission’s *Consumer Conditions Scoreboard* (European Commission, 2019). The Consumer Scoreboards, which have been published annually since 2008, present survey data from 30 European countries. They consist of two series, published in alternative years: The Markets Scoreboard, which offers a Market Performance Index (MPI) based on consumer evaluations of approximately 50 consumer markets, and the Conditions Scoreboard, which estimates a Consumer Condition Index (CCI). In both series “trust” is central to the construction of the indexes. The results presented here are based on the latest Consumer Conditions Scoreboard before revisions. The data material was collected by GfK Social and Strategic Research (GfK SSR) between 26 March and 11 May 2018 (European Commission, 2019, Annex I).

In total, 28 037 respondents, living the 27 Member States of the European Union, the UK, Iceland, and Norway, responded to the survey. For 26 of the countries, approximately 1,000 respondents answered the questionnaire, whereas there were 500 respondents for each of the four smallest countries, Luxembourg, Cyprus, Malta, and Iceland.

To be nationally representative, a random sample was drawn from the population 18 years or older. Individuals were contacted via mobile phone and fixed lines in every country. The sampling procedure was set up to achieve a mix of respondents. The sample intake was stratified according to gender, age, and the ownership of a mobile and/or fixed phone.

To ensure that the data were nationally representative, the results for each country were weighted according to age, gender, and type of telephone. The average EU28 result was weighted according to the population size of the countries. In the multivariate analyses, populations are unweighted, but probable sample biases at country levels are controlled for by including education (higher education = 1), gender, and age in the analyses.

### Operationalization of Main Variables

The traditional way to measure generalized trust (World Values Survey and the European Social Surveys) is by asking, “Generally speaking, would you say that most people can be trusted or that you can’t be too careful in dealing with people?” (e.g., Nannestad, 2008). In this paper, the main dependent variable—a proxy variable measuring *consumers’ generalized trust* at individual level—is based on the extent to which respondents expect *suppliers to respect their consumer rights*. So, if a consumer thinks that suppliers normally respect consumer rights, the response is interpreted as generalized trust/social capital which facilitates the consumer’s role. The respondents participating in the 2019 Consumer Conditions Scoreboard were presented with the following statement:

- In general, retailers and service providers respect your rights as a consumer.

The respondents were asked to rate their response to the statement on a scale from “strongly agree” (5), “agree” (4), “disagree” (2), to “strongly disagree” (1). To complete the scale, “DK/NA” (Don’t Know/No Answer) was given the mid-value (3).

Respondents’ rating of this variable at the *country level* is treated as a proxy for *consumer conditions*, based on citizens’ aggregated perceptions of suppliers’ reliability. The variable is only included in the analyses at either the individual or national level.

Because history, funding, and organizational models concerning consumer policy differ considerably among the 30 countries, it is difficult to find an accurate and comparable measure that captures consumer institutions’ efficiency and the quality of consumer support and protection. To my knowledge, such a measurement has yet to be developed.

However, consumers’ aggregated evaluations, based on their experiences, offer a simple way to estimate institutional performance. National levels of trust in consumer authorities are continuously calibrated by the consumers, based on their experiences (Berg, 2014). In this paper, citizens’ aggregated trust in consumer authorities’ ability to protect consumer rights is used as a proxy for *consumer authorities’ efficiency and performances* in a country, understood as the authorities’ power to enforce consumer rights. This trust was measured by responses to the following statement:

- You trust public authorities to protect your rights as a consumer.

Individuals rated their response on a scale from “strongly agree” (5), “agree” (4), “disagree” (2), to “strongly disagree” (1). “DK/NA” was given the mid-value (3).

Implicitly, the operationalization of *consumer authorities’ efficiency* presupposes that consumers’ trust reflects the performances of consumer institutions, which, of course, need not always be the case. The trusted is not always trustworthy, and trust is vague and unstable. Nevertheless, few if any procedures can provide rock-solid and true measures of consumer authorities’ and institutions’ performances (Berg, 2014). Within each country, the inhabitants have access to the same consumer markets and relate to the same consumer authorities. Thus, the aggregated estimates for each country are considered to give robust assessments of consumer authorities’ performances.

The other independent variable at the country level*—citizens’ financial capability*—is based on households’ aggregated reported financial situation, determined by respondents’ completion of the following statement:

- Thinking about your household’s financial situation, would you say that making ends meet every month is… “very difficult” (1), “fairly difficult” (2), “DK/NA” (3), “fairly easy” (4), or “very easy” (5)?

At individual level, the analyses control for *individual financial situation* (1–5) and *individual trust in consumer authorities* (1–5), as well as for *gender* (women = 1), *age* (four cohorts), and *education* (higher education = 1).

### Analyses

The 28 037 survey respondents considered consumer conditions in a total of 30 different countries. To distinguish *high-trust countries* from *low-trust countries* according to citizens’ trust in consumer authorities and in suppliers, a benchmark was set at 70% positive answers. This benchmark was chosen based on the visible pattern that emerges when countries are ranked according to the two trust variables. The benchmark was used to distinguish three appropriate groups of countries. *High-trust countries* are those in which more than 70% of respondents expressed trust (choosing “strongly agree” or “agree” to the statements above). *Low-trust countries are those in which* less than 70% express trust in response to both trust statements. The remaining countries were classified as *high trust in suppliers only*.

The Consumer Conditions Scoreboard data provide the opportunity to repeat and compare multivariate results at individual levels from several countries. Accordingly, it is possible to determine if the same mechanisms are present in all countries or if different mechanisms are present in different countries. Multivariate analyses at the individual level within each country make it possible to investigate and compare how *individual trust in consumer authorities*, *financial situation*, *age*, *gender*, and *education* affect *individual generalized trust in retailers and providers*. To facilitate the interpretation of the results, only significant, standardized beta-coefficients are presented. The multivariate analysis split on countries only compares the results from the 26 contries representing approximately 1.000 respondents each. Luxembourg, Malta, Cyprus, and Iceland are not analysed separately because each represents only 500 respondents, which impacts significance levels. These respondents are included, however, in the aggregate analyses, in which respondents living in *high-trust countries* (*N* = 11.011) are compared to those in lower-trust countries (*N* = 17.026).

The final analysis combines and distinguishes between variables at individual and country levels. The purpose of the multilevel analysis is to illustrate the interplay between institutional- and individual-level impacts, with the goal being to depict how institutionalized distrust (measured by *consumer authorities’ efficiency*) at the country level may affect *generalized trust* at individual level.

The multilevel technique SPSS Mixed was discarded and replaced by traditional path analysis because path analysis provides a more plausible model, separating between independent, intermediate, and dependent variables, and thus can show how institutional variables at the country level work through variables at the individual level before affecting the dependent variable (at the individual level).

To simplify the model, the group variables of *age*, *gender*, and *education* are only included (controlled for) in the last step of the path analysis. However, if they were included in Step 1, the main result would be the same.

In the multivariate analyses, simple linear regression models are applied. The measurement levels of the variables included are not perfectly precise (ordinal level), and in interpreting the results, only robust patterns are commented on. The material contains more than 28 000 respondents, meaning that almost every estimate will show significant results (*p* < 0.01) when all respondents are included. The main purpose of the multivariate analyses is to discover and to report robust patterns.

## Results

### How Do Trust in Consumer Authorities and in Retailers and Providers Vary Across Europe?

**Fig. 2:**
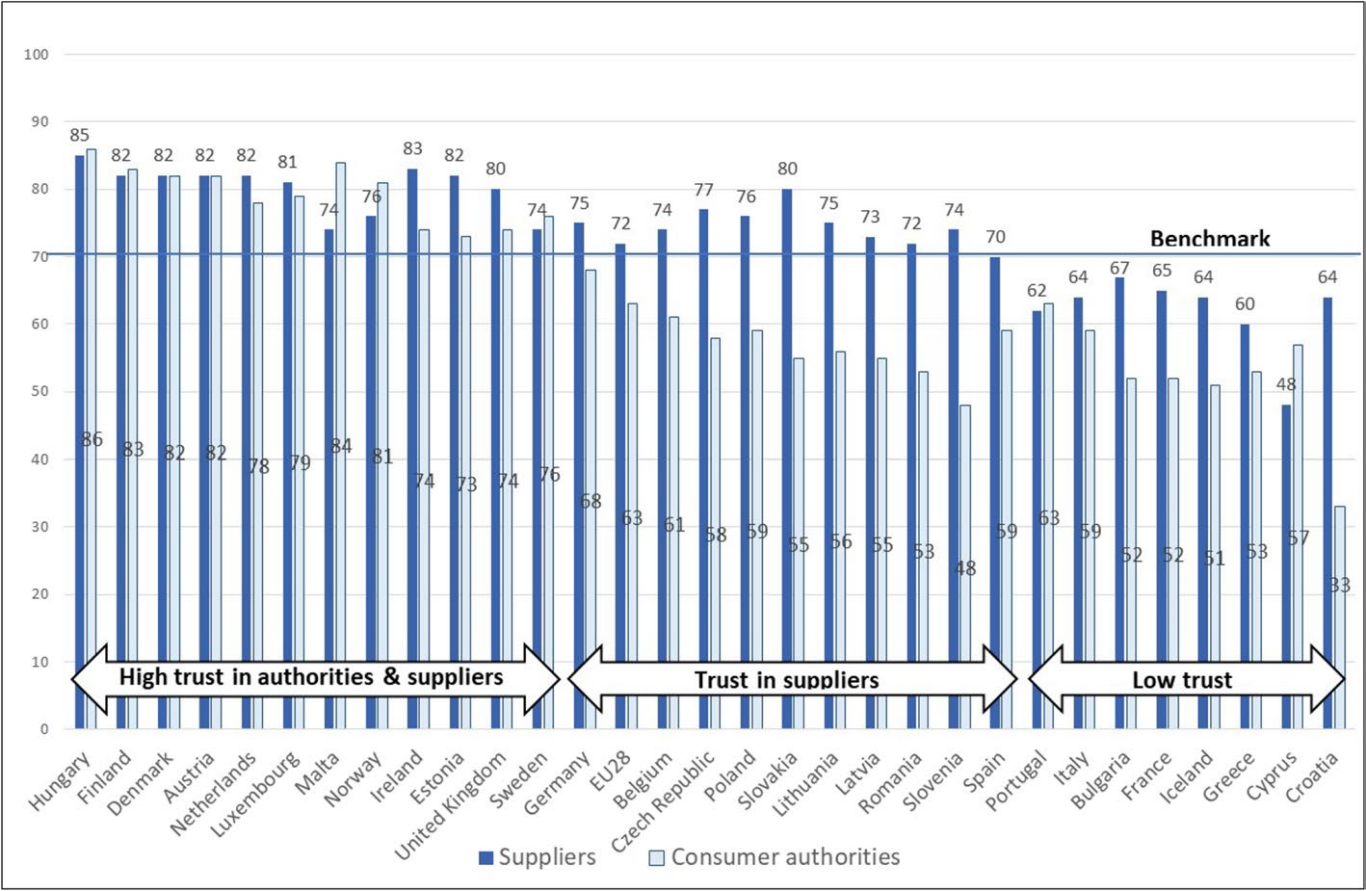
European countries ranked according to citizens’ levels of trust. Percent. (*N* = 28.037)

The columns in Fig. 2 show percentages of those who responded “strongly agree” or “agree” to statements reflecting trust in public authorities and suppliers, respectively. The countries are ranked according to level of citizen trust. What does the pattern of trust reveal?

The variations in trust presented in Fig. 2 support findings from two earlier waves of the Scoreboard material, which also reported large differences in levels of trust across Europe (Berg, 2014; Nessel, 2021). Trust in suppliers varies considerably (diff. 37%) but to a lesser extent than does trust in public authorities (diff. 53%).

To distinguish between countries’ trust levels, a benchmark for high trust was set at 70 percent (see “Methods”). The horizontal arrows illustrate how the 30 European countries included in the analyses can be categorized according to citizens’ trust. Twelve countries can be categorized as *high-trust countries*, with 86% of respondents (Hungary) to 73% (Estonia) reporting trust in consumer authorities and 85% (Hungary) to 74% (Malta and Sweden) stating that they trust suppliers. Ten countries—as well as the European Union average—seem primarily to place trust in the market forces. Within this group of respondents, 80% (Slovakia) to 70% (Spain) report trust in suppliers, whereas only 68% (Germany) to 48% (Slovenia) say they trust their authorities. Eight countries are categorized as *low-trust countries*, with only 63% (Portugal) to 33% (Croatia) saying they trust their authorities and 67% (Bulgaria) to 48% (Cyprus) reporting trust in their suppliers. Notably, and without exception, the countries with consumers who expressed high trust in consumer authorities also deem consumer conditions to be safe, as measured by the extent to which suppliers are viewed as respecting consumer rights. More precisely, no data correspond to the fourth possible category: “high trust in consumer authorities” combined with “low trust in suppliers.”

The countries included in the Consumer Scoreboard data draw on varied political and market histories. These histories have likely shaped market conditions, as well as where consumers place their trust (Putnam, 1993). Berg et al. (2005) find that consumers’ trust in food safety in Denmark and Norway were more likely to rest on their trust in public food control, whereas in the St. Petersburg region, trust in food safety was more likely to depend on trust in market mechanisms. Figure 2 reveals a similar pattern: Respondents living in former communist countries tend to express less confidence in public authorities than do those in other European countries, with the majority of former Eastern Bloc countries falling into the group *high-trust in suppliers only*. Similar patterns are reported by Marozzi (2015): Scandinavians show the highest trust in public institutions, whereas citizens in former communist countries and Southern European countries are less trustful.

The categories and rankings in Fig. 2 are comparable to Austgulen’s (2020) classification of consumer policy regimes. The Nordic and Franco-Roman countries are predominately *high-trust countries*, whereas the Southern and Eastern European countries fall into the categories of *low-trust countries* and *trust in suppliers only*. The Anglo and German countries fit into the upper half of the rankings.

There are exceptions. According to Fig. 2, consumers in Hungary appear to be the most satisfied with their country’s consumer authorities and suppliers. This was somewhat unexpected since new Eastern Member States in general are characterized by lower levels of consumer protection than older Member States (Austgulen, 2020). In search of explanations for the result, consumer policy experts working in different Norwegian consumer institutions as well as in the European Commission were consulted. The experts noted that Hungarian authorities had recently made strong efforts to improve consumer conditions. Also, according to Nessel (2019), who compared consumer policies of 28 EU Member States, Hungary ranked number 4 on the *legal protection index* and number 8 on the *public enforcement index*. One interpretation of the results in Fig. 2 is that Hungary, in comparison to other Eastern Member States, is gaining generalized trust by improving consumer conditions in home markets. However, the Hungarian government’s response to the Russian invasion of Ukraine give reason to problematize their high trust results. According to mainstream Western media (April 2022), Hungary, although an EU country, refuses to sanction Russia if doing so would harm Hungary’s internal economy, e.g., hinder access to Russian gas supplies. This nationalistic policy is closely tied to consumer concerns, such as the cost of energy. Is it possible that Hungary’s consumer policies are not only consumer-friendly, but also part of a nationalistic strategy to gain citizens’ trust?

Another unexpected result shown in Fig. 2 is the placement of France, a country characterized by strong, not weak, institutions, among the *low-trust countries*. This result is also contested by Nessel’s (2019) findings with respect to policy, which ranked France as second best on the *public enforcement index* and as number 3 on the *legal protection index*. The consumer experts who were consulted expressed puzzlement at France’s low ranking. It is possible that a serious market or national event, occurring just prior to the collection of the consumer conditions data, influenced the French responses to statements about trust.

As expected, the Nordic countries stand out as *high-trust countries*. Unlike most other countries, the consumer authorities in the Nordic countries are ranked as equally high or higher in trust than are suppliers. One exception is Iceland, placed among the *low-trust countries*, probably as a result of the banking crisis that led to the country’s total financial collapse in 2008.

### Demographic Variations

In addition to citizens’ self-reported *financial situation*, Table 1 lists the other independent variables included in the analyses that follow. The countries are ranked according to their trust levels as illustrated in Fig. 2.

**Table 1:** Citizens’ self-reported financial situation, age, gender, and educational level. Percent. (*N* = 28.037)

	Financial situation		Age cohort 34–54	Gender Women	Higher education
	Easy	Difficult			
Total	64	34	35	52	43
Hungary	47	52	36	53	29
Finland	88	12	31	51	52
Denmark	87	12	34	51	26
Austria	82	17	35	51	37
Netherlands	83	15	34	51	45
Luxembourg	74	23	38	50	24
Malta	53	38	32	50	35
Norway	87	11	35	50	60
Ireland	69	29	39	51	65
Estonia	69	30	33	54	42
UK	73	24	34	51	57
Sweden	89	10	33	50	47
Germany	81	17	34	51	38
Belgium	63	35	34	51	40
Czech Rep	65	35	37	51	26
Poland	62	36	34	52	41
Slovakia	50	48	36	52	40
Lithuania	58	42	33	55	63
Latvia	52	45	33	55	45
Romania	63	35	36	52	36
Slovenia	66	33	36	51	45
Spain	47	48	39	51	40
Portugal	41	55	36	53	28
Italia	50	49	36	52	37
Bulgaria	46	52	35	52	47
France	61	38	34	52	51
Iceland	80	17	31	50	41
Greece	31	67	36	52	48
Cyprus	45	53	33	52	53
Croatia	46	51	33	52	44

Table 1 reveals large differences in the *financial situation* of citizens living in different European countries. Respondents living in *high-trust countries* (see Fig. 2) are much more likely than others to report that their financial situation is easy. In the Nordic countries, as well as in Austria, the Netherlands, and Germany, as many as 80% or more of respondents described their household’s *financial situation* as easy. With the exception of Iceland, less than 12% of respondents in Scandinavia (Finland, Denmark, Norway, and Sweden) reported that their household’s *financial situation* was difficult, compared to as much as 67% in Greece and over 50% in Hungary, Portugal, Bulgaria, Cyprus, and Croatia.

The *gender* distribution for the entire sample is 48% men and 52% women. Only Lithuania and Latvia deviate more than ± 2% from this average with 55% women. For the entire sample, 35% are in the *age* cohort 34 to 54 years old, with variations by country ranging from 32 in Malta to 39% in Spain. Only five countries deviated more than ± 2% from the 35% average. The figures for *education* varied widely, likely indicating different operationalizations across the countries. For Luxembourg, 24% reported having higher education, whereas the figure was 65% for Ireland. The average for the respondents of all 30 countries was 43% reporting higher education.

According to Table 1, countries’ aggregated levels of trust may be affected by citizens’ *financial situation* but are unlikely affected by *age* or *gender* distributions (small variations). Because of possible operationalization differences, the *education variable* is probably only of interest for analyses within countries.

### Do Fair and Efficient Consumer Authorities Enhance Consumer Conditions?

As already noted, it is not certain that citizens’ evaluations of the consumer conditions in a country are foremost determined by consumer authorities’ efficiency and fairness. Also, citizens’ financial situation, which varies considerably across countries, may affect how people experience and perceive consumer conditions.

Starting at the country level: If *citizens’ financial capability* is controlled for, how does the efficiency of a country’s *consumer authorities* affect citizens’ perception of their *consumer conditions*?

**Fig. 3:**
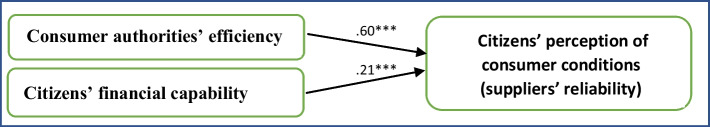
Effects from consumer authorities’ efficiency and citizens’ financial capability on consumer conditions. Aggregated country level. Linear regression. Standardized beta-coefficients (β), *** = *p* < .001. r^2=^ .56 (*n* = 30)

Figure 3 supports the idea that *consumer authorities’ efficiency* has a strong impact on *citizens’ perception of consumer conditions* (β = 0.60***). According to the previous theoretical review, the working mechanism can be described as a disciplining power streaming from consumer institutions’ institutionalized distrust—supporting consumers and trustworthy suppliers, while punishing unserious businesses—resulting in more reliable suppliers and safer consumer conditions, as judged by the consumers.

Figure 3 also indicates that *citizens’ financial capability* influences how suppliers’ reliability was reported (β = 0.21***). This result may reflect that, in the market game, a financially rich population acts as a stronger counterpart to the supply side than does a population with fewer financial resources. And/or, and probably more likely, that consumers with strong financial power are less vulnerable to consumer detriments because they can afford to make a bad buy, as well as to choose quality products, while this is not the case for consumers with less financial power (Berg, 2016).

### Does Trust in Consumer Authorities Affect Generalized Consumer Trust?

We know that there are large differences among countries in *citizens’ financial capability* (Table 1). Could the result at the country level presented in Fig. 3 indicate that it is foremost the financially rich who, regardless of country, experience safe consumer conditions and therefore report a high level of trust in providers and suppliers? To investigate if this is the case, Table 2 lists the results of 26 multivariate regression analyses on individual levels, controlling for *financial situation*, *gender*, *age*, and *education*. The four smallest countries, due to the small number of respondents, are not included in this comparison.

Preferably, *financial situation*, *age*, *gender*, and *education* should not affect *individual generalized trust*, i.e., people’s considerations of suppliers’ reliability. If such individual characteristics appear to impact people’s considerations of suppliers’ reliability, this may be a sign that different groups are treated differently in consumer markets.

Table 2 lists countries according to their trust rankings as illustrated in Fig. 2. The first two rows, however, distinguish between those respondents living in *high-trust countries*, and the others, who reside in countries with lower trust levels.

**Table 2:** How individual generalized trust in suppliers, within countries, is affected by individual trust in consumer authorities, individual financial situation, age, gender, and education. 26 linear regressions. Significant standardized beta-coefficients (β), *** = *p* < .001 (high-trust countries *n* = 11.011, lower-trust countries *n* = 17.026, country *n*≈1,000)

	r^2^	*Ind. trust in consumer authorities*	*Individual financial situation*	*Age*	*Gender*	*Education*
**High trust countries**	.10	.29***	.03***	-08***	.02*	
**Lower trust countries**	.10	.27***	.10***	-07***	.02*	.01*
Hungary	.12	.33***		.10***		
Finland	.08	.20***		-.18***		
Denmark	.14	.35***		-.06*		
Austria	.09	.26***	.11***	-.09**		
Netherlands	.11	.32***				
Norway	.15	.34***		-.15***		
Ireland	.13	.31***	.07*	-.09**	.09**	
Estonia	.07	.22***		-.08**		
UK	.13	.33***	.09**		.09**	
Sweden	.08	.20***		-.16***		
Germany	.08	.25***	.09**	-.10***		
Belgium	.09	.26***	.12***			
Czech Rep	.10	.24***	.11***	-.08**		
Poland	.10	.31***	.10***			
Slovakia	.04	.20***				
Lithuania	.13	.30***		-.14***		
Latvia	.09	.26***	.11***			
Romania	.13	.35***				
Slovenia	.08	.24***	.10**			
Spain	.09	.26***	.07*	-.10**		
Portugal	.10	.24***	.12***	-.10**		
Italia	.14	.29***	.08**	-.18***	-.07*	
Bulgaria	.10	.28***		-.13***		
France	.08	.26***	.10***	-.08		
Greece	.08	.22***	.10**	-.09***		-.07*
Croatia	.13	.34***	.10***			

In all countries surveyed, *individual trust in consumer authorities* has a strong impact on *individual generalized trust in suppliers* (from β = 0.20*** in Finland, Sweden, and Slovakia, to β = 0.35*** in Denmark and Romania). As shown in Table 2, this is the case both in *high-trust countries* characterized by fair and effective consumer authorities and in countries with lower trust levels. *Age* shows a reasonably stable pattern, as people in 16 out of 26 countries seem to grow more sceptical as they get older. Perhaps this is because they become more experienced as consumers or perhaps, although less likely, because older consumers in these countries are more likely than younger consumers to be discriminated against in markets. One exception to this stable age pattern is people in Hungary, a high-trust country; there, older people are more trusting than younger, indicating that Hungarian markets to a greater extent meet the needs and expectations of the older than the younger consumers, when the variables *financial situation*, *education*, *gender*, and *individual trust in authorities* are held constant.

Particularly noteworthy is the two first rows of Table 2, which contrast people living in countries with fair and effective consumer authorities to those in countries with less effective authorities. They indicate that *individual financial situation* has no significant impact on how respondents rate suppliers’ reliability in *high-trust countries* characterized by fair and effective consumer authorities, as judged by their fellow citizens. In countries in which fellow citizens consider consumer authorities’ fairness and efficiency to be lower, however, financially poor people are less likely than the rich to trust suppliers.

This pattern is reflected in the separate country analyses as well. Only three out of 10 *high-trust countries*, compared to 12 out of 16 *lower trust countries*, reveal significant effects from *individual financial situation* on *generalized trust in suppliers*. Among the *high-trust countries*, only within Austria, Ireland and UK do consumers seem to be treated differently in the marketplace depending on their financial status. *Individual financial situation* bears no correlation with the level of trust in suppliers reported by consumers in the Scandinavian countries, Hungary, and the Netherlands. This was also the case in Estonia, a country pointed to by experts as leading among Eastern European countries in adjusting to Western European consumer policy standards. It seems, then, that fair and effective consumer institutions may protect against unequal treatment of consumers according to their financial situation.

Without commenting on all results presented in Table 2, the pattern supports the idea that *individual generalized trust in retailers and providers* is related to *individual trust in consumer institutions*. Within countries, *gender* and *education* seldom show significant effects. The exceptions are the UK and Ireland, where female consumers tend to be more trusting than males, and Italy, where males tend to be more trusting than females, all other variables kept constant.

The main result at this point in the analysis is that fair and effective consumer institutions lead to improved consumer conditions as well as to more equal treatment of consumers, regardless of their financial situation.

### Are Fair and Effective Consumer Authorities Drivers of Generalized Trust?

**Fig. 4:**
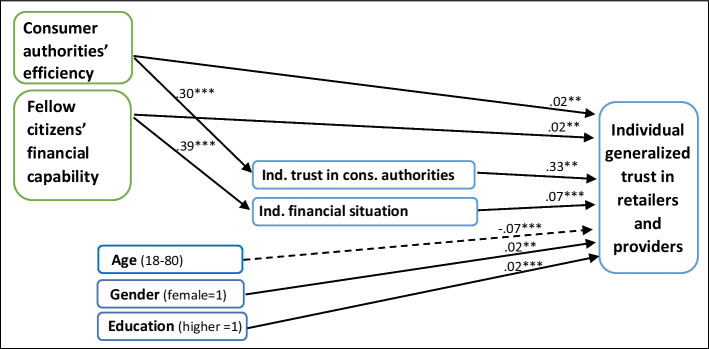
Is Individual generalized trust in retailers and providers affected by consumer authorities’ efficiency? Three linear regressions. Significant standardized beta-coefficients (β), *** = *p* < .001. r.^2=^ .08, .15, .14 (*N* = 30, 28.037)

Eventually, the last figure (Fig. 4) illustrates how *consumer authorities’ efficiency*, working through *individual trust*, affects *individual generalized trust in retailers and providers*. Variables at the country level are outlined in green, and variables at the individual level in blue.

The multilevel path analysis presented in Fig. 4 supports this paper’s main hypothesis: Consumer institutions’ fairness and efficiency is a substantial driver for generalized trust in the markets. However, almost the entire effect that originates with *consumer authorities’ efficiency* is channelled through *individual trust*. It is interesting, logical, and reasonable that *individual trust in consumer authorities* (β = 0.33***) conquers *consumer authorities’* more objective *efficiency* as it is judged by respondent’s fellow citizens (β = (0.02 + (0.30*0.33) = 0.12**). It only means that an individual’s experiences in the marketplace are much more significant in affecting a person’s trust in a supplier than are consumer authorities’ fairness and reliability as it is perceived by his/her fellow citizens. As an example, two persons living under the same consumer policy regime will—easily—judge consumer conditions differently, if one of them accidentally bought a bad product from a less-than-trustworthy supplier, and the other did not have such an experience.

Similarly, the results in Fig. 4 also shows that, within a country, *fellow citizens’ financial capability* has a slightly smaller effect (β = (0.02 + (0.39*0.07) = 0.05**) than does *individual financial situation* (β = 0.07**) on *individual generalized trust in suppliers*. The strong effect from *fellow citizens’ financial capability* on the intermediate variable *individual financial situation* only demonstrates that a person’s salary not only depends on him/herself but quite a lot on the national economy.

As expected, *gender* and *education* have minor effects on individual trust in suppliers to respect consumer rights. *Age*, however, shows the expected negative effect; older people, who have had more consumer experiences than younger people, appear to be less trusting than those who are younger (β =  − 0.07**).

## Conclusion and Discussion

The foregoing analyses, based on survey data from 28 037 respondents living in 30 European countries, indicate that fair and effective consumer authorities are strong drivers of better performing markets, measured by safe consumer conditions. Further, the multilevel path analysis supports the main hypothesis suggesting that fair and effective consumer authorities enhance generalized trust in the markets. As a by-product, fair and effective consumer institutions also contribute to more equality in the markets.

The results support Berggren and Jordahl (2006) who claim that well-functioning markets can be expected to give rise in trust. The analyses also support the institutional centred approach that claims that government institutions and policies create, channel, and influence trust and social capital (Fukuyama, 2002; Marozzi, 2014; Rothstein & Stolle, 2008; Rothstein, 1998). Finally, the results support the view that individual trust in institutions, or system trust (Luhmann, 1979, 1988), should be added to social networks (Coleman, 1988), as determinants of an individual’s social capital.

Trust happens at individual level. It is logical—and reflected in the multilevel analysis—that people’s assessments of their consumer authorities are based on their individual experiences in the marketplace. Thus, although this paper treats generalized trust as a dependent variable, we can sense virtuous circles including both individual and societal levels. First, fair and effective consumer authorities’ enforcement of consumer rights (institutionalized distrust) fosters reliability among suppliers and safe conditions for consumers. Second, safe consumer conditions are paving the ground for good consumer experiences, giving consumers reasons to trust (Braithwaite, 1998). And third, according to theory (Coleman, 1990; Marozzi, 2014), generalized trust is valuable as social capital at the individual level and highly valuable as societal capital at the national level, promoting welfare (Elster, 1989; Fukuyama, 1995), prosperity (Algan & Cahuc, 2013), and democracy (Putnam, 1993, 2000; Sztompka, 2010). Accordingly, to invest in fair and effective consumer institutions promoting safe consumer conditions appears to be a good national, as well as European, strategy to increase valuable generalized trust among residents.

However, there is one problem related to safe consumer conditions and trust. As argued in the paper, trust can be passivating. Hence, it is imaginable that too safe consumer conditions could result in many naïve consumers making unreflective choices (Berg, 2004, 2008). One key question, then, is how much risk should consumer authorities seek to eliminate to protect consumers without passivating the consumers by trust? Should consumers, rather than be protected, instead be better informed? Or are consumers already overloaded by information (Berg & Gornitzka, 2012)? How can consumers be encouraged to make reflective choices, to compare products, and, perhaps most important, to give voice and complain (Hirschman, 1970) when they have reasons for that? These are complex consumer policy questions. One approach, supported by insights from behavioural economics, would be for consumer authorities to facilitate informed consumer choices by requiring suppliers to present choice architectures that help consumers to make choices in their own interests (Thaler & Sunstein, 2008).

It is also a reason to believe that peoples’ opinions about how safe, fair, and well-organized the markets are likely influence their considerations about, and trust in, other societal institutions with which they may have limited personal experiences (Berg, 2014; Holm, 1998). Because nearly all people have experiences with markets, but only some have experiences with the police, courts, hospitals, social security, etc., it is probable that peoples’ opinions on how safe, fair, and well-organized the markets are will influence their considerations about, and trust in, other societal institutions. More precisely, people may assume that market conditions reflect how their society—including its governance bodies—is functioning.

### Further Research

The main hypothesis investigated here deserves to be studied further, from different angles and using other data. In particular, the development of a better measure of a country’s investment in, and the performance of, its consumer institutions is needed.

The Markets Scoreboard also reveals large differences in consumer trust *between* markets *within* the same country (European Commission, 2018). Hence, it is important to understand better the variations in trust between markets and how such variations relate to specific characteristics inherent in the markets, consumers’ practices, or institutions’ performances. Eventually, it would be very interesting to study how generalized trust—originating from market interactions between buyers and sellers—may extend to, and interplay with, other societal institutions, such as police, courts, hospitals, social security, and governments.
